# Recent Advances in 3D Printed Electrodes – Bridging the Nano to Mesoscale

**DOI:** 10.1002/advs.202411951

**Published:** 2025-02-11

**Authors:** William J. Scheideler, Jisun Im

**Affiliations:** ^1^ Thayer School of Engineering Dartmouth College Hanover NH 03755 USA; ^2^ School of Engineering University of Warwick Coventry CV4 7AL UK

**Keywords:** 3D architected electrodes, 3D printing, 3D sensors, additive manufacturing, electrochemical devices

## Abstract

3D architected electrodes offer inherent physicochemical advantages for energy storage, conversion, and sensing. 3D printing methods such as stereolithography and two photon polymerization are uniquely capable of fabricating these architected electrodes with a high degree of geometric complexity impossible to achieve with other methods at the mesoscale (10 µm–1 mm). The material set for 3D printing traditionally is focused on structural materials rather than functional materials suitable for electronic and electrochemical applications. In this review the fundamental challenges are considered for transforming 3D printed materials into conductive, multifunctional electrodes suitable for electrical and electrochemical devices by printing nanocomposites, infusing molecular precursors and post‐processing these structures via carbonization. To understand the design of 3D electrodes toward their use in both sensors and electrochemical devices such as catalysts, this review summarizes recent advances in hierarchical design of porous metastructures, the engineering of mass transport and electronic transport in 3D structures, and the application of high‐throughput materials design by machine learning and artificial intelligence. These emerging approaches to 3D electrode design and architecture promise to expand the capabilities of additive manufacturing beyond structural materials and bring its advantages to bear on modern devices such as sensors, batteries, supercapacitors, and electrocatalysts.

## Graphical abstract

3D architected electrodes offer inherent physicochemical advantages for energy storage, conversion, and sensing. This review summarizes recent advances in fabrication and design of 3D electrodes, the engineering of mass transport and electronic transport, and the application of high‐throughput materials design by machine learning. Finally, it consider how to transform 3D printed materials into multifunctional electrodes for sensing and catalysis.

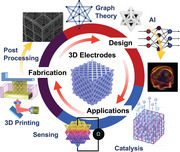

## 1 Introduction

3D architected materials offer properties inaccessible in bulk, for example, spatially and temporally tailored responses to various physical stimuli,^[^ ^1^ ^]^ hierarchically engineered properties over length scales spanning the micro and mesoscale (µm–mm),^[^ ^2^ ^]^ and an amenability to customization via *on‐demand* additive manufacturing (AM) methods. The traditional focus of 3D architected materials was on mechanical property design, but a range of applications to energy materials,^[^ ^3^ ^]^ tissue engineering,^[^ ^4^ ^]^ biosensing,^[^ ^5^ ^]^ and optics^[^ ^6^ ^]^ is emerging. AM, specifically 3D printing, is the primary approach for fabricating these 3D architectures because traditional substrative methods are unable to reach the necessary resolution or allow the free‐form design of self‐supporting complex structures. As AM has advanced in its scope and resolution, the design of 3D printed functional materials is a natural extension toward new application spaces. 3D printed functional materials could advance beyond traditional bulk processing methods, accessing an immense 3D geometric design space for enabling integration of miniaturized sensing^[^ ^7^ ^]^ and energy technologies^[^ ^8^ ^]^ at the mm and sub‐mm scale. Toward this end, this review focuses specifically on 3D printed electrodes incorporating conductive materials.

The key engineering challenges for developing 3D printed electrodes concern their design and fabrication. Addressing these challenges requires considering the 3D scaling of electrical and electrochemical properties and the influence of mass transport in these porous structures. The initial objective is to learn how to efficiently navigate the near‐infinite 3D design space to derive suitable 3D electrode geometries for a given application. The next related challenge is how to fabricate 3D electrodes, which calls for understanding how these processing methods (times, temperatures, and modalities of annealing) are intrinsically linked to the material properties. Conversion from liquid phase precursor inks or photopolymer precursors requires energy inputs, typically in the form of heat to drive these reactions. Various approaches, for example, from nanocomposite formation to carbonization, are available to transform 3D printed polymers into functional electrodes, with comparative advantages for devices requiring a combination of electrical, electrochemical, and structural characteristics.

Efficiently leveraging 3D printed electrodes requires that we take a pragmatic view of both their unique capabilities and limitations. We should first analyze the inherent advantages of architected 3D electrodes, versus random materials (e.g., foams) and composites made by bulk processing. Recent review papers addressed the advantages and challenges of 3D printed hierarchical structured electrodes and summarized their performance compared to traditional planar disk type electrodes for energy storage devices, such as batteries and supercapacitors.^[^ ^9^, ^10^ ^]^ The 3D printed structures discussed in this review facilitate precise geometric control over physical properties in multiple domains, extending the potential for application specific design of electrodes. This review will discuss several target applications in sensing and energy that utilize the unique geometric complexity of 3D printed parts, specifically their ability to facilitate gas and liquid phase mass transport through their pore structures. A related focus of this paper is on how to overcome the inherent limitations to the patterning resolution of photopolymerization – we consider how to design electrodes that get around this submicron barrier to bridge the nanoscale and mesoscale features of 3D printed structures. For example, using nanomaterials, how might we design hierarchical materials that use the micro and mesoscale architectures but retain the high surface area of nanoscale materials.

**Figure 1:**
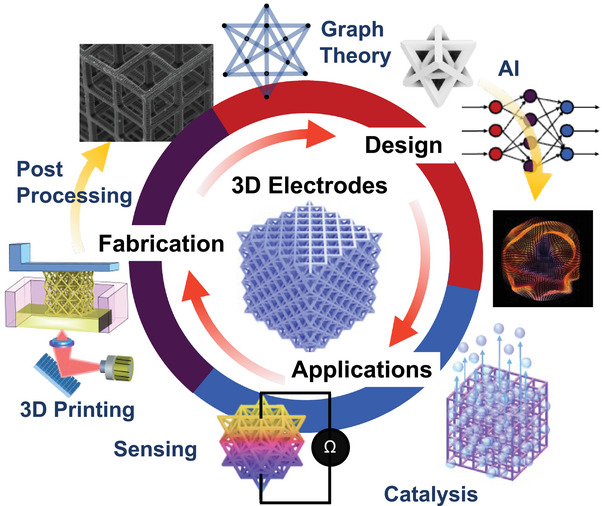
Schematic depicting topics of active research in fabrication, design, and applications of 3D printed electrodes.

This review of 3D printed electrodes first introduces the leading methods for 3D printing (Section 2) and considers their resolution and comparative advantages. We summarize the strategies available for extending these methods to conductive materials and analyze the direct 3D printing of composites versus post‐processing by high temperature carbonization or deposition of conductive coatings. The design of 3D structures and their electrical and electrochemical properties are then discussed, along with emerging approaches to accelerate design with machine learning and artificial intelligence. Section 3 builds off this background to review the application of 3D printed electrodes in both sensors and energy systems. Finally, we consider the challenges and opportunities for 3D printed electrodes, specifically the Multiphysics design and the potential for deeper 3D integration with microelectronics. The scope of this review is graphically summarized in **Figure** **1**.

## 2 Results and Discussion

### 2.1 Overview of Microscale 3D Printing Methods

**Figure 2:**
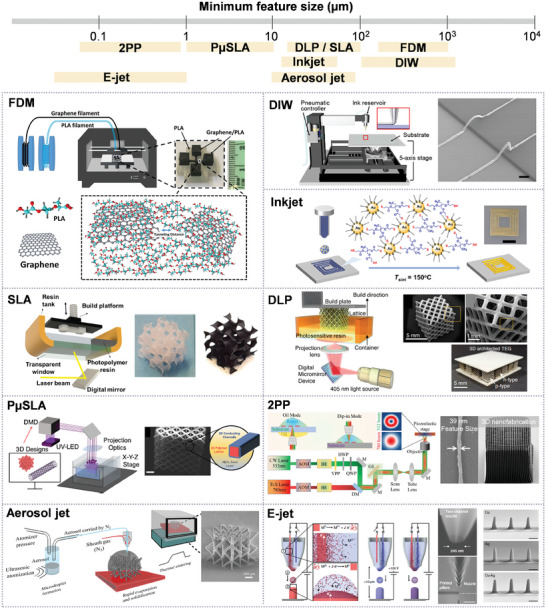
Leading AM techniques for fabricating 3D structured electrodes from nano to meso‐length scales. Reproduced with permissions from refs. [21, 24, 26, 33, 34, 35, 41, 47], Copyright 2022, Materials Today, Copyright 2022, Wiley, Copyright 2022, Cell Press, Copyright 2020, Wiley, Copyright 2021, Elsevier, Copyright 2022, Wiley, Copyright 2023, ACS, Copyright 2023, Wiley.

In this section, we summarize the key AM technologies used for fabricating 3D structured electrodes at nano‐ to meso‐length scales and address their unique capabilities for different materials and resolutions. Recent development in printing processes and materials for each technique is also highlighted. **Figure** **2** provides schematics of this collection of electrode manufacturing methods, depicting the range of length scales that are accessible, from the submicron scale to the mm‐scale. Example 3D structures based on these methods are provided to illustrate the comparative levels of geometric complexity. For these various methods, we have also provided **Table** **1** which reviews the resolution, speeds, layer thicknesses, and post‐processing methods utilized for various 3D printed electronic materials.

**Table 1:** Tabular summary of the resolution, speed, thickness, and post‐processing methods reported for 3D printing of electronic materials.

Printing method	Material	Minimum feature size	Printing speed	Layer thickness	Post‐processing	Application	refs.
FDM	Graphene/PLA Carbon black/PLA Copper‐based filament		15–40 mm s^−1^			Electronic components (e.g., resistor, inductor and capacitor)	[12]
	Graphene/PLA					Electron tunnelling based devices	[14]
	Graphene/PLA				Chemical treatment	Biosensor	[15]
	CNT/PLA	500 µm			Carbonization	Li‐ion battery	[61]
	Graphene/PLA Carbon black/PLA Copper‐based filament	500 µm	15–30 mm s^−1^	100 µm	Cu electrodeposition	Inductor	[83]
DIW	LiFePO_4_, Li_4_Ti_5_O_12_, and Ketjenblack active particles‐based inks (η = 10^4^–10^5^ Pa·s at 0.1 s^−1^)				UV curing	Li‐ion battery	[8]
	TiH_2_ particles/acrylate resin	250 µm	1 mm s^−1^	600–900 µm	Pyrolysis	Metallic origami structures	[17]
	Liquid metal (EGaIn)‐polymer composite inks (η = 10^3^–10^5^ Pa·s at 0.1 s^−1^)	210 µm	30 mm s^−1^			Flexible, non‐planar electrode	[19]
	PEDOT:PSS (η = 10^2^–10^5^ Pa·s at 0.1 s^−1^)	30 µm		17 µm	Thermal annealing	Flexible electronic circuit	[20]
	Liquid metal (EGaIn)	1.9 µm	0.1–3 mm s^−1^			3D stretchable electrodes	[25]
	Reduced graphene oxide/CNT inks	400 µm	5–30 mm s^−1^		Electrochemical deposition (Ni‐NiO)	Water splitting	[100]
	Carbon nanofibers/PDMS inks (η = 10^4^ Pa·s at 0.1 s^−1^)	500 µm	6 mm s^−1^		Thermal annealing	Pressure sensors	[109]
	CNT/PDMS inks (η = 10^3 –^ 10^4^ Pa·s at 0.1 s^−1^)	400 µm	5 mm s^−1^		Heat treatment	Piezoresistive sensors	[110]
	CNT/SiNPs/silicon elastomer resins	400 µm	15 mm s^−1^		Heat treatment	Flexible pressure sensors	[111]
	MoS_2_ nanocomposite hydrogel	300 µm	5 mm s^−1^	200 µm		Electronic skin	[112]
Inkjet	Gold nanoparticle‐based nanoink	62 µm	30 mm s^−1^	164 nm	Thermal sintering	Flexible electrode	[29]
	Graphene ink	245 µm	50 mm s^−1^		Thermal annealing	Transistor	[31]
	PEDOT:PSS	100–200 µm		100–300 nm	Solvent treatment	Stretchable interconnects	[33]
SLA	Epoxy/acrylate resin	50 µm	1.3 mm s^−1^	50 µm	Pyrolysis	Sodium ion battery	[3]
	Acrylate resin	100 µm			MXene coating	Tissue‐integrated antibiotic sensor	[5]
	Epoxy/acrylate resin	50 µm		80 µm	Carbonization	3D microstructured electrodes	[57]
	MgO NPs / photoresin	100–200 µm		50 µm	Pyrolysis	Capacitor	[59]
	Photocurable resin	200 µm		100 µm	Electroless plating (Ni)/electroplating (CoNi_2_S)	Supercapacitors	[81]
DLP	Formlabs high‐temperature resin				MnO_x_ surface decoration and pyrolysis	Supercapacitor	[39]
	Acrylate resin	50 µm			Carbonization and thermal evaporation of Sb_2_Te_3_ and Bi_2_Te_3_	Thermoelectric generators	[40]
	Metal salts containing photoresin	100 µm			Calcination	Li ion batteries	[53]
	Ag nanowires / acrylate resin (η = 1300 cP)	300 µm		30 µm	Pyrolysis	3D microsupercapacitors	[54]
	Acrylate resin	26–50 µm	60 µm s^−1^		Pyrolysis	Electrochemical applications	[58]
	Acrylate resin	50 µm		25 µm	Pyrolysis	Li ion batteries	[60]
	UV curable resin			25 µm	Carbonization	Microbial fuel cell	[62]
	Graphene/silica/acrylate resin			50 µm	Spark plasma sintering	High temperature electronics	[64]
	Acrylate resin	40 µm		50 µm	Hydrogel infusion (Cu, CuNi) / calcination	Metallic micromaterials	[70]
	Nanoclay/acrylate resin	50 µm		30 µm	Post UV curing	Tactile applications	[113]
	Ionnogel	5 µm		20 µm		Capacitive sensors	[114]
PµSLA	Photopolymer resin	10 µm			Metal oxide coating	Multimodal sensors	[26]
	Acrylate photoreactive resin	2 µm		10 µm	Electroless plating	Enzymatic biofuel cells	[41]
	Acrylate resin	10 µm		20 µm	Electroless plating	Zn ion batteries	[56]
2PP	HAuCl_4_ / acrylate resin	78 nm	0.1‐5 mm s^−1^		Pyrolysis	Plasmonics and flexible electronics	[42]
	Ni‐acrylate photoreactive resin	25‐100 nm	4‐6 mm s^−1^	150 nm	pyrolysis	3D nanostructured metals	[43]
	Ag salt / acrylate resin	319 nm	15 mm s^−1^			3D nanostructured composites	[44]
	Zn nitrate/acrylate photoresin	250 nm	1 mm s^−1^		Calcination	Electromechanical devices	[69]
	PEDOT:PSS/acrylate resin	100 nm		100 nm		Humidity sensors	[115]
Aerosol Jet	Ag nanoparticle inks (η = 1.5 cP)	20 µm			Thermal/photonic sintering	Microelectrodes	[46]
	Pd, Cu, Ag, Au	85 nm	8–100 nm ^−1^s			3D plasmonic metamaterials	[48]
	Ag nanoparticle ink (η = 1.5 cP)	10–50 µm			Thermal sintering	Li ion batteries	[86]
	Ag nanoparticle ink	10–15 µm	5–10 µm s^−1^		Thermal sintering	Biosensor	[108]
E‐Jet	PEDOT:PSS	0.3–20 µm	0.02–1 mm s^−1^		Thermal annealing	Metal interconnects and electrodes	[51]
	Ag, Cu, Ag/Cu	150 – 250 nm	1–2 µm s^−1^			3D nanostructured electrodes	[52]

Fused deposition modelling (FDM) is one of the most common AM processes due to its affordability for cost‐effective rapid prototyping. It utilizes melting and extruding a polymer feed filament through a heated nozzle to deposit material onto a build platform to create a 3D object layer by layer. Due to the relatively large nozzle diameter ranging from 0.25 to 0.4 mm, the minimum feature size of the FDM method is ≈250 µm.^[^ ^11^ ^]^ Most of commercially available feed materials are thermoplastic polymer filaments, including polylactic acid (PLA) and acrylonitrile butadiene styrene co‐polymer (ABS). Conductive polymer composite filaments such as graphene/PLA (e.g., Black Magic 3D), carbon fiber/PLA (e.g., ProtoPasta), and copper‐based polymer (e.g., Electrifi) composites are commercially available for fabricating 3D electronic parts, whose volume resistivities are 0.6 Ω∙cm, 30 Ω∙cm, and 0.006 Ω∙cm, respectively.^[^ ^12^ ^]^ Despite their relatively poor electrical conductivities, due to the low volume fraction of conductive fillers that affect the percolating networks of conductive nanomaterials within the polymer matrix,^[^ ^13^ ^]^ these materials offer opportunities in applications such as resistors^[^ ^12^ ^]^ and electrochemical devices,^[^ ^14^, ^15^ ^]^ often after post‐processing methods such as thermal, chemical and electrochemical treatments.

Direct ink writing (DIW) is also an extrusion‐based AM technique, where a liquid phase material is extruded through a nozzle onto a build platform and solidified by solvent evaporation or UV curing between printed layers to create a 3D object. DIW is compatible with a wide range of materials, as long as the ink exhibits appropriate rheological properties such as suitable viscosity in the range of 10^2^–10^6^ centipoise (cP) at a shear rate of ≈0.1 s^−1^, yield stress shear thinning behavior, and viscoelastic properties.^[^ ^16^ ^]^ 3D electronic components were fabricated by DIW using various conductive materials such as metals,^[^ ^17^ ^]^ metal alloys,^[^ ^18^ ^]^ liquid metals,^[^ ^19^ ^]^ and conducting polymers.^[^ ^20^ ^]^ Due to its versatility for material selection, DIW has become a powerful tool for fabricating 3D structured electrodes for energy storage devices.^[^ ^21^, ^22^, ^23^ ^]^ The minimum feature size of the DIW method is typically sub‐millimeter depending on the nozzle size. Smaller nozzles can improve printing resolution, but they require higher extrusion pressure and longer build time to prevent nozzle clogging.^[^ ^24^ ^]^ Park, et al. demonstrated that a minimum line width of 1.9 µm of liquid metal (EGaIn, 75.5% gallium and 24.5% indium alloy by weight) was directly printed through the nozzle with inner diameters of 5 to 40 µm by coordinating the operation of the pneumatic pressure (on/off) and the movement of translation stages.^[^ ^25^ ^]^

Inkjet printing involves generating droplets ejected from a nozzle by pressure pulses, typically produced through thermal or piezoelectric methods. By simultaneously controlling the ejection process and the position of the printhead or substrate, droplets of materials are deposited onto a build platform to create a part layer by layer.^[^ ^27^ ^]^ The feature size of inkjet printing depends on the ejected liquid droplets with the volume of several picoliters, which limits the minimum feature size of printed dots or lines to tens of micrometers.^[^ ^28^ ^]^ A wide range of conductive materials have been developed for inkjet printing processes to print conductive electrodes. Printing metal nanoparticles/flakes‐based conductive inks followed by sintering is one of the most prevalent strategies for fabricating highly conductive printed electronics.^[^ ^29^, ^30^ ^]^ Carbon‐based nanomaterials, such as graphene^[^ ^31^ ^]^ and carbon nanotubes,^[^ ^32^ ^]^ and conducting polymers^[^ ^33^ ^]^ are also demonstrated by inkjet printing process for various electronic applications including transistors and sensors. Multiple printing steps depositing consecutive layers can be used to produce 2.5D and 3D structured electrodes, to a certain degree. However, this process requires support material to print overhang structures because of the low to medium viscosities of inkjet inks.

Vat polymerization is an AM process that utilizes a light source to initiate photopolymerization in a photoreactive resin to fabricate 3D structures layer by layer. Depending on the light source and curing mechanism, it can be further categorized to stereolithography (SLA), digital light processing (DLP), projection micro‐stereolithography (PµSLA), and two photon polymerization (2PP, known as two photon lithography). SLA, DLP, and PµSLA utilize the single photon absorption and the photopolymerization occurs on the surface of the photoreactive resin that only allows building 3D structures layer by layer.^[^ ^34^ ^]^ SLA uses a UV laser to cure a photoreactive resin by point‐to‐point tracing the geometry of each layer on the surface of the resin vat, offering high resolution and a very smooth surface finish.^[^ ^34^ ^]^ The minimum feature size ranges from 25 to 50 µm depending on the laser spot size and the increments at which the laser beam is controlled.^[^ ^35^ ^]^ DLP uses a projected light source to cure an entire layer at once. This is achieved using a digital micromirror device (DMD) that projects the image of the layer, with each pixel in the image corresponding to a tiny mirror that reflects light onto the resin. DLP also provides a similar minimum feature size (10–50 µm) to that of SLA,^[^ ^36^ ^]^ offering high resolution. The build speed of DLP is generally much faster than SLA because it cures an entire layer at once rather than tracing it point‐by‐point. The PµSLA system exposes a photoreactive resin with top‐down illumination through projection optics using a 405 nm UV light emitting diode and offers the printing resolution down to 0.6 µm by adjusting the magnification of the projection lens.^[^ ^11^, ^26^ ^]^

2PP uses a femtosecond near‐infrared laser that provides sufficient peak intensity at a low average laser power to enable the polymerization of the photoreactive resin by absorbing two photons simultaneously. The polymerization process occurs only within the focal volume (known as a voxel) of the pulsed light through a high numerical aperture objective lens within a photoreactive resin, which enables achieving a high photon density near the focal point to fabricate sub‐micron structures. With precise control of laser intensity, scan speed, and suitable objective lens, the sub‐micron feature size can be achieved. For high‐precision and high resolution below 100 nm, the stimulated‐emission‐depletion (STED) inspired 2PP was developed, where the effective excitation volume was physically reduced using an continuous inhibition (or depletion) laser beam along with a femtosecond excitation laser.^[^ ^37^, ^38^ ^]^

The most well‐established strategies for fabricating conductive 3D structures in the nano to mesoscale range using vat polymerization include 1) 3D printing of polymer scaffolds followed by carbonization^[^ ^39^, ^40^ ^]^ or metallization such as electroless plating^[^ ^41^ ^]^ and sputtering,^[^ ^26^ ^]^ 2) simultaneous photopolymerization and photoreduction of metal salt precursor within photoreactive resin,^[^ ^42^, ^43^ ^]^ and 3) polymerization of conductive particle‐loaded photoreactive resins.^[^ ^44^, ^45^ ^]^ Post‐processing steps such as sintering and pyrolysis are required to achieve highly conductive nano/microstructures.

Aerosol jet uses a focused sheath of gas to transfer the aerosolized microdroplets to fabricate highly intricate microscale 3D networks such as microscaffolds/microlattices with nearly fully dense truss elements without the use of any supporting materials.^[^ ^46^ ^]^ This method allows the transfer of a wide range of feedstock materials with a viscosity of up to 1000 cP to be printed, which includes inks and dispersions containing nanoparticles of metals, metal oxides, polymers, and other ceramics. Wide standoff distances (1–11 mm) between the nozzle and the substrate allows conformal printing of complex geometrical designs on non‐planar surfaces, e.g., stepped or curved surfaces.^[^ ^47^ ^]^ Saleh, et al. fabricated 3D microarchitectures using silver nanoparticles (AgNPs) dispersed in an aqueous ethylene glycol solution as a source of building material.^[^ ^44^ ^]^ They demonstrated 3D Ag microlattices consisting of truss elements as thin as 20 µm in diameter, with open void sizes ranging from 100 µm to 1 mm. Jung, et al. demonstrated various 3D metallic structures with minimum feature sizes down to hundreds of nanometers using charged aerosol jet method.^[^ ^48^ ^]^ The printing process does not require polymers or inks. Instead, ions and charged aerosol particles, generated by spark discharge, are directed onto a dielectric mask with an array of holes that floats over a biased silicon substrate. The hole containing mask is similar to the conventional 3D printer nozzle, but the feature size of printed structures is much smaller than the size of the holes due to the electrostatic focusing.

Electrohydrodynamic jetting (E‐jet) can generate very fine ink droplets by applying an electric field between a conducting nozzle and substrate.^[^ ^49^ ^]^ An electric field applied to the nozzle tip causes mobile ions in the ink to accumulate near the surface at the tip of the nozzle. Coulombic repulsion between the ions introduces a tangential stress on the liquid surface that deforms the meniscus of the droplet into a conical shape known as a Taylor cone.^[^ ^50^ ^]^ When the electrostatic stress overcomes the surface tension, ink droplets are ejected from the cone.^[^ ^49^ ^]^ E‐jet system uses an electric field to pull the droplets from the small nozzles with inner diameters ranging from 100 nm to a few micrometers, offering high‐precision and high‐resolution pattern printing at the nanometer and micrometer length scales.^[^ ^27^, ^51^ ^]^ Reiser, et al. introduced electrohydrodynamic redox printing, which makes it possible to directly produce polycrystalline metal 3D structures using metal ions‐loaded solvent droplets without further processing after printing.^[^ ^52^ ^]^ When these droplets contact the substrate, the transfer of electrons from the substrate reduces the ions to form a metallic deposit. Reiser et al. demonstrated Cu patterns with line widths below 100 nm.

### 2.2 Processes for Transforming 3D Printed Electrodes

**Figure 3:**
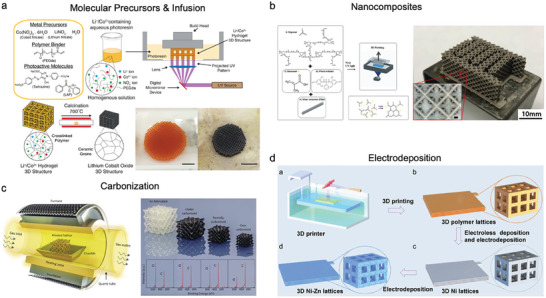
Methods for post‐processing of 3D electrodes. a) Molecular precursor and infusion (reproduced with permission from ref. [53], Copyright 2021, Wiley), b) nanocomposite resins (reproduced from ref. [54], Copyright 2018, ACS), c) carbonization of polymers (reproduced with permission from ref. [55], Copyright 2022, Cell Press), and d) electrodeposition on polymeric 3D scaffolds (reproduced from ref. [56], Copyright 2021, Wiley).

There are inherent challenges to apply 3D printing toward conductive electrodes. 3D printing methods have previously been engineered for prototyping and for structural materials. The materials that work well for these methods, including photopolymers and thermoplastic precursor materials for 3D printing, are typically not conductive. This leads to few predominant strategies for 1) incorporation of conductive filler materials (e.g., nanocomposites) or molecular precursors to functional materials into 3D printable resins, 2) post‐processes such as carbonization at high temperatures to convert 3D printed polymers into conductive phases, or 3) functionalization of 3D parts via coating with conductive materials by liquid phase electrodeposition or vacuum deposition. These strategies are summarized in **Figure** **3**.

#### 2.2.1 Carbonization of Polymers

Carbonization of 3D printed polymers can be controlled to yield conductive electrodes that retain the microscale features printed by SLA or DLP and amplify the resolution of SLA / DLP 3D printing. Carbonization processes for converting the polymer resin to a conductive graphitic phase consist of slow heating to a temperature sufficient to decompose or pyrolyze the polymer chains.^[^ ^57^ ^]^ Additional efforts have been devoted to controlling the porosity of carbonized lattice structures at various length scales. For example, Steldinger, et al. have shown that porogen molecules can be incorporated into the resin and later removed to template pore formation at the 100′s nm scale for carbonized monoliths.^[^ ^58^ ^]^ This approach preserves the high resolution (15 µm features are demonstrated) of the 3D printed structures as well as their mechanical strength. The advantage of this porosity control is the achievement of high surface area up to 2200 m^2^ g^−1^. In a similar approach, Kudo, et al. have shown that magnesium oxide (MgO) nanoparticles can be incorporated into photoresins to template pore formation after MgO removal via soaking of the structures in hydrogen chloride (HCl).^[^ ^59^ ^]^ The authors present a hierarchical pore structure comprised of 3D designed pores, macropores at the 5 µm level, and nanoscale pores that amplify the surface area.

Carbonization results in a dramatic change in the mechanical characteristics as the polymer phase is transformed to a ceramic phase, though multiple studies have shown that the ceramic carbonized electrodes can be engineered to avoid brittle failure. For example, Narita, et al. showed that carbonized DLP printed lattice structures can attain high specific strength (101 kN·m kg^−1^ comparable to 6061 Al).^[^ ^60^ ^]^ This study also showed, via x‐ray diffraction (XRD) and Raman spectroscopy, the nanoscale structure consisting of a mixture of both glassy carbon and stacked graphitic layers also termed turbostratic graphene. Slow multi‐step heating to avoid bubble formation during gas removal as well as slow cooling allowed the formation of intact and high strength carbonized 3D lattice structures. The mechanical strength as well as the dimensional stability of 3D structures during carbonization can also be aided by the incorporation of 0D, 1D, and 2D carbon nanomaterials, as recently described by Park, et al.^[^ ^61^ ^]^ Park, et al. report in this work that the multiscale, aligned networks consisting of carbon nanotubes (CNTs) embedded in the carbonized polymers have the effect of reducing charge transfer resistance and improving the compressive Young's modulus. In this case, carbonization serves to *join* the carbon nanomaterial elements to improve the structural integrity and electrical conductivity. Finally, an additional, complementary approach to achieve both high strength and ductility in carbonized lattice electrodes is to utilize lower temperature carbonization to preserve a combination of sp^2^ and sp^3^ bonded carbon with the presence of graphene‐like flakes, as shown by Surjadi, et al.^[^ ^55^ ^]^ Unconverted polymer chains in partially carbonized structures can serve to block crack propagation and enhance the ductility of the mixed ceramic / polymer phase. One potential tradeoff, however, in partial carbonization is likely to be lower electrical conductivity due to the insulating nature of the remaining polymer phase.

The carbonization process itself can influence the electrical and electrochemical properties of the resulting structure. Bian, et al. have recently shown that carbonization of SLA printed 3D lattices at 800 °C in N_2_ atmospheres can also result in N_2_ doping of the resulting porous carbon anodes utilized for microbial fuel cells.^[^ ^62^ ^]^ N‐doping, the authors argue, can aid the formation of catalytically active sites to enhance carbon electrode performance. Arrington, et al, report that carbonization of imides such as 3D printed polyimide lead to high mechanical integrity and low shrinkage with electrical conductivity on the order of 1–3 S cm^−1[^ ^63^ ^]^ Carbonization of composites with high conductivity fillers such as graphene can also produce highly conductive (1000 S cm^−1^) ceramic phases such as silicon carbide (SiC) when utilizing higher carbonization temperatures (1200 °C).^[^ ^64^ ^]^ An advantage of SiC ceramics is that they can also provide stable electrical properties during operation at elevated temperatures (up to 600 °C). Carbonization of 2D structures has also become a common method used in the flexible electronics field via laser processing of laser‐induced graphene conductors.^[^ ^65^ ^]^ The rapid laser carbonization process is many orders of magnitude faster than bulk carbonization, occurring at the millisecond time‐scale,^[^ ^66^ ^]^ however, this process must still retain the substrate to enable mechanical stability. The intersection of laser‐induced graphene processing with 3D printed structures^[^ ^67^ ^]^ offers a promising extension of these rapid methods to form structural electrodes for uses in energy and sensing applications. Additionally, post‐processing of carbonized structures via joule‐heating of the conductive element could engineer the surface morphology and surface energy.^[^ ^68^ ^]^

#### 2.2.2 Molecular Precursors and Liquid Phase Infusion

Molecular precursors integrated into the 3D printed resin or infused after printing provide an alternative route to transform 3D printed structures into functional electrodes. For example, Yee, et al. in 2019 demonstrated 3D printing of microarchitected metal oxides (zinc oxide, ZnO) via incorporation of metal nitrate salts (zinc nitrate) into poly(ethylene glycol) diacrylate (PEGDA) precursor resins for two photon polymerization.^[^ ^69^ ^]^ Calcination of these structures (500 °C) resulted in nanocrystalline ZnO structures with submicron feature sizes following the dimensional reduction upon annealing. By a similar method, Yee, et al. have also demonstrated the incorporation of hygroscopic metal salts such as lithium and cobalt nitrate into aqueous PEGDA resins, forming lithium cobalt oxide (LCO) lattices from an intermediate hydrogel via DLP 3D printing.^[^ ^53^ ^]^ Heating at 700 °C to form ceramic LCO structures yielded functional cathodes in lithium (Li)‐ion batteries. The ability to control the metal oxide composition in this approach could yield additional opportunities for various porous ceramics by use of metal nitrate salts. In 2022, Saccone, et al. introduced a closely related method known as hydrogel infusion additive manufacturing (HIAM) for transforming polymer microlattices into 3D metal structures (e.g., Ag, Ni, Cu, CuNi, W‐Ni, etc.).^[^ ^70^ ^]^ This approach relies on first performing a solvent exchange in aqueous metal nitrate solutions to infuse metal ions into the polymer, followed by a sequence of calcination in air (700 °C) to form a microstructure consisting of metal oxide grains that are ultimately reduced to pure metal via high temperature forming gas annealing (900 °C).

Inspired by the HIAM method, we have recently demonstrated a polymer infusion additive manufacturing (PIAM) using commercial acrylate photoresins as the precursor to generate conductive core‐shell electrodes with graphitic struts coated in a high surface area mixed metal / metal oxide coating.^[^ ^71^ ^]^ Though PIAM and HIAM share similar metal nitrate precursor chemistries, our PIAM method utilizes N_2_ annealing rather than calcination in air, allowing the retention of the underlying polymer struts and conversion via carbonization. Subsequent reductive annealing allows control over the relative metal / metal oxide phase distribution. Similar to HIAM, these methods result in a significant dimensional reduction, supporting the printing of high‐resolution strut lattices even with cost effective DLP and SLA equipment with modest capabilities.

#### 2.2.3 Nanocomposite 3D Electrodes

The electrical properties of 3D printed polymers can be engineered via addition of conductive fillers including carbon nanomaterials, conducting polymers, and metal particles. This approach offers the potential for directly printing conductive electrodes without significant post‐processing; however, the tradeoff is that fillers scatter and absorb light in stereolithography processes, reducing photosensitivity and degrading the resolution. For example, multiwalled carbon nanotubes (MWCNTs) can be used to form conductive composites via SLA but the 3D printing process requires higher UV curing doses and longer exposure times due to the parasitic absorption of the CNT fillers.^[^ ^72^ ^]^ The other major effect of fillers is that they can substantially increase the viscosity of stereolithography resins. This can be problematic if the resin is no longer able to effectively flow out of the cavities of complex 3D printed lattice structures.^[^ ^59^ ^]^ Loading with 1D nanomaterials such as CNTs can have appreciable effects on the viscosity of acrylate resins even at relatively low concentrations such as 0.25% wt.^[^ ^73^ ^]^ Maintaining lower viscosity in the case of conductive polymer (poly(3,4‐ethylenedioxythiophene):polystyrene sulfonate (PEDOT:PSS) / PEGDA composites) can be achieved using appropriate surfactants to preserve 3D printability of composite resins.^[^ ^74^ ^]^

The electrical performance of 3D printed conductive nanocomposites is highest for metallic fillers and moderate for carbon nanomaterial fillers. MWCNT composites achieve conductivity in the range of 10^−4^ S cm^−1[^ ^73^ ^]^ to 0.04 S cm^−1[^ ^75^ ^]^ and 0.08 S cm,^−1[^ ^72^ ^]^ likely limited by the density of the percolation network and the resistive junctions formed by resin between CNTs. This performance level has been sufficient to demonstrate devices such as capacitive sensors.^[^ ^72^ ^]^ Metal nanoparticles can offer 3D printed composites with higher conductivity, for example, 1000 S cm^−1^ as printed for composites formed from multiple conductive fillers (AgCu microflakes, Ag NPs, and CNTs).^[^ ^76^ ^]^ In this study by Tsai, et al., the purpose of the multiple fillers including CNTs is to prevent the settling of the microscale metallic fillers. This result reveals the necessary balance between the stability of the suspension (which favors high viscosity) and the printability of SLA resins (which favors low viscosity). Ag NP formation in situ via the conversion of metal salts to nanoparticles was reported by Sciancalepore, et al. though these low density composites did not offer sufficient conductivity for electrode applications.^[^ ^77^ ^]^ Additional methods for controlling alignment of conductive fillers offer the ability to enhance their conductivity at lower concentrations of fillers. For example, Wang, et al. report significantly enhanced conductivity enabled by acoustic assembly of Ag NPs.^[^ ^78^ ^]^ Though not for the specific purpose of boosting electrical conductivity, Melchert, et al. recently showed that carbon fibers and boron nitride (BN) sheets could be aligned via an acoustophoresis method to create 3D printed structures with anisotropic thermal conductivity.^[^ ^79^ ^]^ Similar methods have shown the alignment of anisotropic 2D nanomaterials using electrical fields to create locally conductive regions.^[^ ^80^ ^]^ These methods for alignment and 3D “patterning” of conductive versus insulating layers could be one step toward the goal of achieving 3D devices with new functionality.

#### 2.2.4 Coating via Electroplating and Surface Modification

Electroplating and electroless deposition offer attractive strategies to post‐process 3D printed polymer structures at the micro and mesoscale. These techniques offer the advantage of using well‐known plating chemistries that have often been optimized for conformal coating of microscale features in the microelectronics industry. Electrodeposition also affords the ability to form various microscale film morphologies to add surface area or other structural features to 3D printed parts. Insulating 3D printed polymers require electrodeposition rather than electroplating. Electrodeposition of Ni has been employed in multiple studies to form a conductive seed layer for subsequent depositions onto periodic 3D lattices. Song, et al. showed Ni deposition was conformal on 3D printed polymer lattices, coating the internal facets with only minor microscale wrinkles.^[^ ^81^ ^]^ Zhang, et al. report microscale SLA printed lattice structures with electroless deposited Ni layers as well as Ni‐Zn layers for use in Zn batteries.^[^ ^56^ ^]^ Electroless deposition on 3D printed polymers, however, first requires surface treatment to improve their wettability and afford uniform deposition on their internal facets to reach a resistivity of ≈45 µΩ·cm. Electroless deposition of Ni has also been employed by Su, et al. in their recent report of 3D printed full cells for water splitting.^[^ ^82^ ^]^ Their structures utilized both electroless Ni coatings of their octet lattices as well as subsequent coatings of catalytically active nickel‐iron(oxo) hydroxide to improve activity.

Electroplated Cu can transform 3D printed polymers into highly conductive electrodes suitable for integration of microelectronics. This can be accomplished by initially 3D printing conductive materials using conductive filaments^[^ ^83^ ^]^ to allow direct one‐step plating. An additional benefit highlighted by Kim, et al. was the reduction in surface roughness of 3D plated parts fabricated through FDM. Additionally, this work showed that electroplated Cu films could render 3D printed parts to be solderable. Lazarus, et al. have also shown that through dual material extrusion it is possible to selectively plate onto individual traces to form circuits on 3D printed structures with low resistivity on part with that of traditional electroplated Cu films.^[^ ^84^ ^]^ This selective formation of conductive electrodes is essential for future 3D printed electronic systems integrating many components at the circuit level. Selective electroplating can also be programmed via SLA resins loaded with Ni nanoparticles, as shown by Credi, et al.^[^ ^85^ ^]^ Although these various demonstrations of selective electroplating have shown structures at the mm‐scale, the chemistry of these methods could likely allow for high‐resolution, high‐density interconnects in 3D if appropriate multimaterial manufacturing method can be applied.

#### 2.2.5 Tradeoffs of Direct 3D Printing versus Post‐Processing

Direct printing of electrodes would be ideal compared with multi‐step post‐processing, but there are tradeoffs to both methods. Post‐processing methods decouple the phase transformations and engineering of the electrochemical and electrical properties from the 3D printing process itself – the advantage of this is that high resolution can be prioritized. Polymer 3D printing has been optimized to yield complex structures and allow for a comparatively low manufacturing cost. Direct 3D printed metals by microscale methods are generally comparatively slow for serial methods relying on DIW or aerosol jet printing.^[^ ^86^ ^]^ In the case of stereolithography printed polymer structures for post‐processing, high performance photopolymers can be selected and no compromises must be made in terms of the resin chemistry and rheology (ultimately the case for resins incorporating conductive fillers or other precursors to functional conductive materials). Post‐processing through carbonization also facilitates the engineering of nanoscale pore structures^[^ ^58^, ^62^, ^86^ ^]^ that are inaccessible for most 3D printing methods and engineering of other surface morphologies tunable for applications requiring high surface area such as electrochemical devices.

Another fundamental factor differentiating post‐processing versus direct 3D printing is the engineering of the bulk versus surface of 3D printed structures. Direct 3D printing generally results in formation of relatively homogeneous compositions and microstructures of the bulk and surface. Post‐processing methods such as electrodeposition and electroplating, however, engineer the surface specifically. This can be advantageous for devices desiring targeted placement of active materials on the interfaces where the reactions take place, such as the case of water splitting full cells^[^ ^82^ ^]^ or the formation of supercapacitors.^[^ ^81^ ^]^ Carbonization does, however, present an opportunity to achieve bulk rather than surface engineering through conversion of the polymer phase to a ceramic phase. Finally, an ongoing challenge for post‐processed 3D electrodes is how to locally control electrical and electrochemical properties. Programming electrodeposition reactions via multimaterial printing represents a promising approach to this.^[^ ^84^ ^]^

### 2.3 Design of 3D Printed Electrode Architectures

3D printed electrodes constitute a large potential geometric design space covering a range of length scales and architectures unachievable for bulk processing or subtractive processing methods. The electrical and electrochemical characteristics of 3D electrodes depend on balancing requirements for efficient transport of both electrons and ions. This typically translates to designs that can simultaneously control both the surface area and the connectivity of the individual structural elements. This section will consider the argument for such ordered, lattice‐type periodic metastructures based on a comparison with stochastic structured porous materials such as foams and compare the variety of 3D periodic geometries that are available. 3D lattices are optimal from many points of view, particularly for high resolution 3D printing processes, since the pore structure can facilitate resin flow, allow for uniform cross‐linking, and use far less material than structures with 100% infill. Lattice structures allow lightweight free‐standing electrodes to be formed by various printing processes and during post‐processing the porous structure can aid in managing stress from high temperature processing and mass loss. Finally, an important motivation for using 3D lattices is that their hierarchical geometries can balance multiple engineering design considerations such as conductivity, strength, and weight. Toward this goal, we review recent developments in the applications of machine learning and artificial intelligence to the acceleration of 3D design and property optimization, noting potential opportunities for novel electrode design.

#### 2.3.1 Comparing Ordered Metastructures versus Random 3D Porous Electrodes

Electrode porosity is a critical feature for many applications requiring high surface area for energy storage, sensing, and catalysis, but traditional bulk material processing cannot offer the same versatility for fabricating various porous or cellular 3D geometries. Open cell foams can be fabricated by a variety of methods^[^ ^87^ ^]^ such as gas injection, foaming of metal melts, sintering of particles, electrodeposition on sacrificial templates, or dealloying. These approaches can yield open or closed cell foams useful in various applications. Metal foams have thermal and electrical conductivities determined by their pore structures. However, a fundamental challenge of randomly porous materials is the inherent variability, particularly when considering properties in microscale and mesoscale structures rather than bulk. For designing microscale devices for energy and sensing, random property variation and anisotropy present an inherent challenge for achieving optimal performance. A useful review of the theoretical properties of cellular porous materials by Ashby in 2005^[^ ^88^ ^]^ compares properties of metal foams as a function of their pore structure. Ashby explains the theoretical of these structures in terms of the topology / connectivity of the pores and the pores shapes, with the relative density as the essential metric. As described in this work, the main distinction of random porous foams is that their random network structure yields compliant, bending deformation dominated structures with low stiffness. Microlattices with ordered structures, however, can be designed with both high porosity and high stiffness through the use of stretching mode structures.^[^ ^89^ ^]^ Ashby also notes the potential for low dielectric constants of foams, and this has been exploited for lattices as well.^[^ ^90^ ^]^

**Figure 4:**
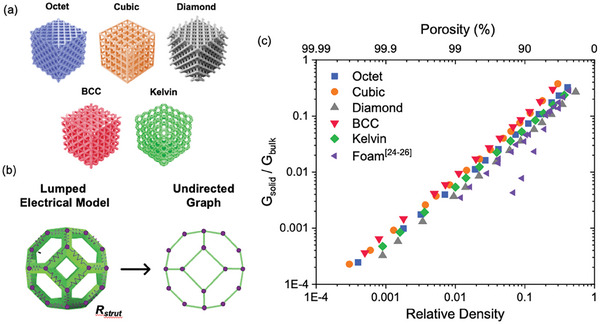
a) 3D renders of common strut lattices, b) graph theory representation of 3D strut lattices as 3D resistive networks, and c) graph theory computed normalized conductivity of 3D strut lattices normalized by bulk conductivity as a function of relatively density (weight). (Reproduced with permission from ref. [91], Copyright 2023, Wiley).

In our recent work,^[^ ^91^ ^]^ we have shown that periodic structures can retain higher conductivity than random structures while offering higher porosity. This makes ordered 3D microlattices attractive structures for lightweight devices and achieving high gravimetric energy density in applications such as supercapacitors and batteries. Different 3D microlattice structures (octet, cubic, body‐centered cubic, kelvin, etc.) offer different performance in terms of electrical, electrochemical properties, and mechanical properties, with microlattices having beams aligned in the direction of current flow offering higher conductivity per weight (**Figure** **4**). The unit cells of body‐centered cubic (BCC) and simple cubic lattices are notably simpler than octet lattices and Kelvin lattices, lacking the cross‐bracing and stretching modes of deformation that would otherwise strengthen the mechanical response. The BCC and simple cubic lattices have beams oriented parallel to the path of current flow from one face to another, resulting in the highest conductivity per weight and per relative density. These optimal electrical characteristics could help to optimize their performance, for example, in terms of gravimetric energy density in energy storage devices. These differences in the electrical properties were similarly observed experimentally for conductive carbonized lattices.^[^ ^91^ ^]^

One of the primary reasons for periodic structures to have electrical advantages over random networks is that the electrical properties of random structures tend to follow the percolation theory^[^ ^92^ ^]^ with conductivity varying exponentially as a function of the relative density. For example, percolation theory predicts power law trend for properties such as electrical and thermal conductivity as a function of relative density. Electrical conductivity exponents have been observed ≈1.55–1.75 for metal foams.^[^ ^92^ ^]^ This means that at low densities the conductivity drops substantially compared with ordered lattices electrical properties^[^ ^91^ ^]^ and also could cause greater variability compared with the comparatively deterministic assembly of 3D printed lattice structures.

#### 2.3.2 Selecting 3D Lattice Metastructures

There are a multitude of 3D lattice structures available for forming electrodes, including families such as strut lattices, surface‐based lattices, and honeycomb (2.5D) lattices. These structures have respective advantages in their relative isotropy or anisotropy of mechanical and other physical properties. Among the most commonly used structures due to their printability and balance of properties, strut‐based lattices are three‐dimensionally periodic arrays of unit cells comprised of rods joined at nodes at various angles. Various common strut lattices can be derived from the set of Bravais lattices, for example, simple cubic lattices, BCC lattices, and face centered cubic lattices (FCC). Other common strut lattice systems include Voronoi, octet, and Delaunay, as shown in Figure 4. Kelvin lattice structures excel in energy absorption due to their structure with bending dominated deformation of the alternating square and hexagonal faces of their unit cell.^[^ ^93^ ^]^ Octet lattices formed with additional cross‐bracing are known for their stiffness due to their stretching dominated deformation modes.

Surface‐based lattices offer an alternative to strut lattices that can avoid the stress concentrations found at the nodes where struts intersect. Triply periodic minimal surfaces (TPMS) such as gyroids have also found a variety of applications as 3D lattice structures. These structures are defined by their zero mean curvature, their non‐intersecting qualities, and their periodic repeating structure. For biological applications, for example, 3D printed tissue scaffold materials, TPMS structures can also offer ideal pore connectivity for bone regeneration.^[^ ^94^ ^]^ Planar, 2.5D lattices like honeycomb lattices are used in certain applications due to the additional flexibility in fabrication though they do not provide 3D control of some properties due to their limited dimensionality.^[^ ^95^ ^]^

The electrical properties of these 3D lattices vary significantly from one type to the next and as a function of their relative density.^[^ ^91^ ^]^ Though more complex unit cells such as octet tend to favor high stiffness, these structures include multiple cross‐bracing beams that contribute little to enhancing electrical conduction in a single direction. Electrical conductivity per weight (or at a given relative density) tends to favor lattices with simpler unit cells (simple cubic, for example, or x‐type body centered lattices). These lattices exhibit the highest conductivity for a given relative density due to the alignment of their beams in a single direction. However, octet lattices and kelvin lattices can also pose advantages for engineering 3D structures with higher surface area for a given unit cell size. We have shown in recent work that the performance of these lattice structures in devices such as supercapacitors corresponds closely with their predicted normalized conductivity.

#### 2.3.3 Design of Hierarchical 3D Electrodes–Advantages for Sensing and Energy Applications

Hierarchical approaches can leverage the 3D design flexibility of AM to enable macroscale new device designs but also enhance performance via engineering of micro and nanoscale morphologies that are beyond the limits of AM methods. Research in hierarchical mechanical metamaterials has shown that fractal‐like repeating self‐similar units can facilitate higher mechanical stiffness^[^ ^96^ ^]^ for metals and can offer improved resilience and recovery after mechanical loading even at extremely low relative densities of just 10^−4^ to 10.^−3[^ ^97^ ^]^ In electrochemical devices, the advantage of hierarchical 3D structures is that they can offer a combination of macroscale pores but nano and microscale high surface area morphologies suitable for surface reactions.^[^ ^98^ ^]^

A fundamental advantage of ordered hierarchical porous structures is that they can balance the requirements for both electronic and ionic transport, with their pore structures facilitating faster diffusion. In the case of 3D printed battery electrodes, hierarchical structures consisting of strut lattices with porous beams have been demonstrated to enhance specific and areal capacitance significantly.^[^ ^86^ ^]^ Similarly, for applications in electrocatalysts, hierarchical structures with nanoporous surfaces on microporous lattices result in substantially higher electrochemical surface area, enhancing the effective catalyst activity.^[^ ^99^ ^]^ In Zhao, et al.’s recent work in 3D hierarchical lattices for water splitting,^[^ ^100^ ^]^ they compare the 3D network of “parent” and “child” channels to those found in biological systems such as lungs. The branching network, they argue, provides efficient mass transport for electrolytes to facilitate full utilization of microscopic surface area.^[^ ^100^ ^]^

The authors demonstrate the effect of this on both the catalyst activity as well as the electrodeposition process used in fabrication, which requires the hierarchical structure for uniform coating of active catalyst materials onto the internal microscopic surface area.

#### 2.3.4 Machine Learning (ML) and Artificial Intelligence (AI) Powered Design of 3D Lattices

**Figure 5:**
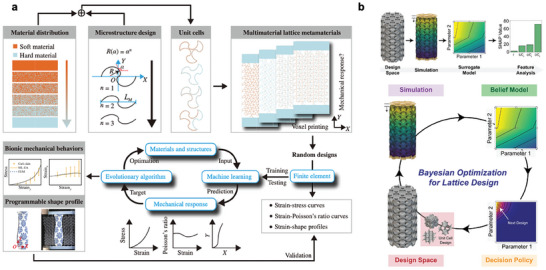
Machine learning (ML) design of 3D structures. a) 3D The digital design and optimization of multimaterial lattice metamaterials by generating the digital voxel models via tuning the material distributions and parametric microstructures, database formation by FEM simulation, training of the ML model mapping the material and structural parameters to the mechanical responses, ML‐EA approach for inverse optimization, followed by voxel printing (Reproduced with permission from ref. [101], Copyright 2022, APS). b) Pipeline for the optimization of lattice structures where the design space is sequentially explored using a Bayesian optimization approach, where designs are selected and virtually tested to iteratively build a belief model and use a decision‐policy to select the next simulation. (Reproduced with permission from ref. [102], Copyright 2024, Springer Nature).

The extremely large design space of 3D porous structures available to AM necessitates efficient means for high‐throughput discovery and simulation of the properties of 3D structures. Machine learning can offer this ability to generate and analyze additional 3D lattice structures. For example, Gongora, et al. have shown that machine learning frameworks utilizing surrogate models (**Figure** **5**) can efficiently explore large parameter spaces covering various 3D lattice structures, accurately predicting their mechanical properties without the need for time consuming full finite element analysis (FEA) simulations.^[^ ^102^ ^]^ In this case, the surrogate model data comes from a limited set of finite element predictions of Young's modulus that sample the parameter space. They additionally show that Bayesian optimization methods can be applied with these methods to derive 3D lattice structures with optimal Young's moduli under certain constraints. Another possibility is to use *generative* machine learning approaches to create additional 3D lattice geometries by selecting from a large set of Bezier curves suitable for SLA printing, as shown recently by Lee, et al.^[^ ^103^ ^]^ Neural networks and genetic optimization were applied to derive structures with optimal mechanical characteristics from a set of tens of thousands of structures while balancing multiple performance metrics. Compared with traditional manual design approaches for 3D structures, deep learning, as shown by Jia, et al., can facilitate more complex designs, for example, lattices that exhibit gradient structures morphing from one lattice type to another.^[^ ^104^ ^]^ These methods can interpolate between multiple unit cells to mix their geometric features and achieve a single structure leveraging the mechanical properties of multiple lattice types. One of the interesting applications of these machine learning methods could be the ability to use AI and ML to generate optimal, non‐identical infills based on lattice structures for different sections of a complex part. For example, denser infill would be optimal for the anticipated high‐stress sections of a part. One could imagine analogous applications to the electrical property design of 3D lattices.

To date, machine learning methods in the field of 3D printing have been focused on mechanical property optimization, though these methods could offer the potential to design specific electrical and electrochemical properties across multiple length scales. One recent work by Yang, et al. has shown the development of carbon microlattice supercapacitor electrodes using machine learning guided optimization of capacitance.^[^ ^105^ ^]^ In this study, the authors SHapley Additive exPlanations analysis to identify the most influential design and processing factors such as electrode thickness and gap dimensions in their optimization of areal capacitance. Beyond this raw experimental optimization of performance, an advantage of these methods is that they can use the power of machine learning to help to solve the *inverse design problems* associated with generating 3D structures exhibiting a specific set of properties,^[^ ^106^ ^]^ for example, a particular areal distribution of conductivity or capacitance. We highlight this possibility for machine learning to accelerate the field of spatially customized 3D printed energy and sensing devices based on microlattice structures. For example, designing electrode materials for energy devices that balance ionic transport and electrical conductivity could be one example, since higher porosity favors electrolyte diffusion while thicker beams and lower pore sizes favor higher electrical conductivity. The ability to grade or vary the lattice structure using machine learning would also be highly useful for the design of sensors integrated into 3D structural parts like a bridge truss^[^ ^107^ ^]^ or the fabrication of sensor arrays with unique responses designed for multiple sensing objectives (for different gaseous analytes, for example).

### 2.4 3D Device Integration for Sensing and Catalysis

#### 2.4.1 3D Printed Sensing Electrodes

AM provides a powerful toolset for developing emerging sensor modalities, allowing for the creation of innovative, high‐performance sensors tailored to meet the specific needs of various applications. The complex, customized 3D structured electrode can enhance the sensitivity by creating larger effective sensing area and functional interfaces. AM allows the realization of electrode architectures that have multiple hierarchical length scales and porosities, which can increase the effective surface area for high sensing performance. This section addresses the advance of the 3D printed sensing electrodes and does not include the development of 3D sensor housing. The 3D printed electrodes for sensor applications have been fabricated by different strategies: 1) customized 3D electrode design architecture either by directly printing 3D electrodes or by printing 3D polymer scaffolds followed by surface activation/modification with sensing materials and 2) new functional sensing materials that can be processable by AM techniques.

**Figure 6:**
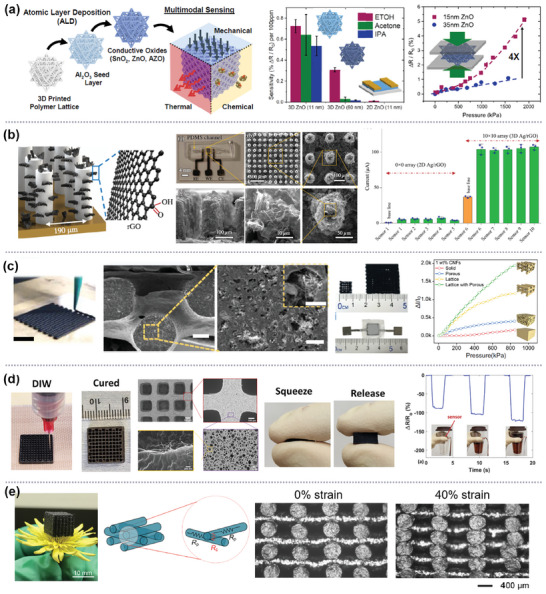
3D sensing electrode architectures with multiscale hierarchical length scales and porosities. a) 3D mesostructured multimodal sensors with nanoscale conductive metal oxides. Reproduced with permission,^[^ ^26^ ^]^ Copyright 2022, Cell Press. b) A nano‐to‐mesoscale biosensing platform based on graphene‐coated electrodes. Reproduced with permission.^[^ ^108^ ^]^ Copyright 2021, Springer Nature. c) Hierarchical in situ filling porous structure for pressure sensor. Reproduced with permission.^[^ ^109^ ^]^, Copyright 2022, Springer Nature. d) Flexible, conductive polymer composites with dual‐scale porosity and piezoresistive sensing functions. Reproduced with permission.^[^ ^110^ ^]^ Copyright 2023, ACS. e) Soft and porous composite‐based pressure sensor with a positive piezoresistive effect. Reproduced with permission Copyright 2020, ACS.^[^ ^111^ ^]^

Huddy, et al. demonstrated the conductive mesoscale lattice structures as multimodal sensors fabricated by printing the polymer lattice using PµSLA and then surface functionalization with metal oxides through conformal atomic layer deposition (ALD).^[^ ^26^ ^]^ In this work, the 3D printed scaffold with ultrasmooth surface allows the deposition of nanoscale functional metal oxide films from conducting (tin(IV) oxide (SnO_2_), ZnO:Al) to semiconducting (ZnO) using ALD with aluminum oxide (Al_2_O_3_) seed layers. The octet lattice was chosen for multimodal sensors in this work due to its high surface area‐to‐volume ratio and the higher theoretically predicted electrical conductivity and thermal conductivity compared with simple cubic or diamond lattices. They demonstrated that the 3D lattices with ultrathin ZnO coatings (11 nm) showed a 100 × enhancement of sensitivity relative to the identical 2D films for ethanol sensing at the ppm level (**Figure** **6**a). It was also demonstrated as a pressure sensor showing a linear relationship between strain and force with 4 × improved sensitivity.

Ali, et al. developed a nano‐to‐mesoscale biosensing platform that can overcome the limits of the conventional electrode architectures to achieve femtomolar level of limit of detection (LOD) for dopamine.^[^ ^108^ ^]^ They fabricated the multi‐length‐scale 3D electrode architecture by printing microscale hollow Ag pillars using aerosol jet and then decorating the pillars with reduced graphene oxide (rGO) nanoflakes (Figure 6b). rGO was chosen in this work for the detection of dopamine due to the electrostatic interaction between positively charged dopamine molecules and negatively charged Ag/rGO micropillar surfaces during the catalytic conversion of dopamine. The sub‐picomolar LOD (500 attomolar) was demonstrated for the detection of dopamine from this sensing electrode integrated with a microfluidic device due to the accelerated electrochemical redox reaction via more effective interactions between dopamine and atomically thin rGO.

Xu, et al. also demonstrated the multiscale hierarchical in situ filling porous piezoresistive pressure sensor fabricated using DIW by extruding carbon nanofibers (CNFs)/polydimethylsiloxane (PDMS) emulsion.^[^ ^109^ ^]^ The in situ filling porous structure was formed by the solidification of CNFs/PDMS emulsion and evaporating of emulsified water at 110 °C, which results in the formation of hierarchical in situ filling porous structure with the CNFs networks embedded in the internal pores with the diameter of 5–10 µm (Figure 6c). This hierarchical geometry significantly increases the conductive contact sites and thus conductive pathways and distributes stress to multilayered lattice and internal porous structure during compression. This results in high sensitivity (4.7 kPa^−1^) and linearity (R^2^ = 0.998) over a broad range of pressure (0.03–1000 kPa) for four layers of face‐centered tetragonal lattice structure printed using the emulsion containing 60 wt.% water and 1 wt.% CNFs.

Abshirini, et al. also used a similar strategy to fabricate highly flexible, ultralightweight, and conductive polymer nanocomposites, consisting of PDMS with MWCNTs, with dual‐scale porosity and piezoresistive sensing functions using DIW.^[^ ^110^ ^]^ In this work, macroscale pores are established by designing structural printing patterns with adjustable infill densities, while the microscale pores are developed by in situ phase separation induced by solvent evaporation during polymer curing. They demonstrated up to 83% tunable porosity by independently controlling the macro‐ and microscale porosity and the flexibility and sensitivity up to 900% and 67%, respectively, while maintaining the mechanical properties (Figure 6d).

Tang, et al. developed a soft and porous composite pressure sensor with tunable piezoresistivity (positive/negative) using DIW to print the ink consisting of CNTs, insulating silica nanoparticles (SiNPs), and a silicon elastomer polymer.^[^ ^111^ ^]^ The piezoresistive coefficient of the printed sensor was tunable by varying the micropore structures and adjustable conductive networks based on the conductive CNTs and insulating SiNPs weight ratio. They studied the piezoresistive mechanism using a printed woodpile structure with pore size in the range of hundreds of micrometers. They found from a simple circuit model that the resistance change was caused by both the decrease in the contact resistance (*R_c_*) due to increased contact areas of the stacked microfibers in the intersection region and the increase in the tunneling resistance (*R_p_*) due to the expansion of the filaments between intersections during compressive deformation (Figure 6e). At the low CNT concentration, the microporous woodpile structure showed a positive piezoresistive effect due to the change in *R_p_* being dominant during deformation because of a small amount of CNTs on the contact surfaces. With an increase in the CNT content, *R_p_* is dominant, but *R_c_* also modulates the output responses, resulting in a linear increase in resistance and the high sensitivity (0.096 kPa^−1^) and a wide sensing range (0–175 kPa).

**Figure 7:**
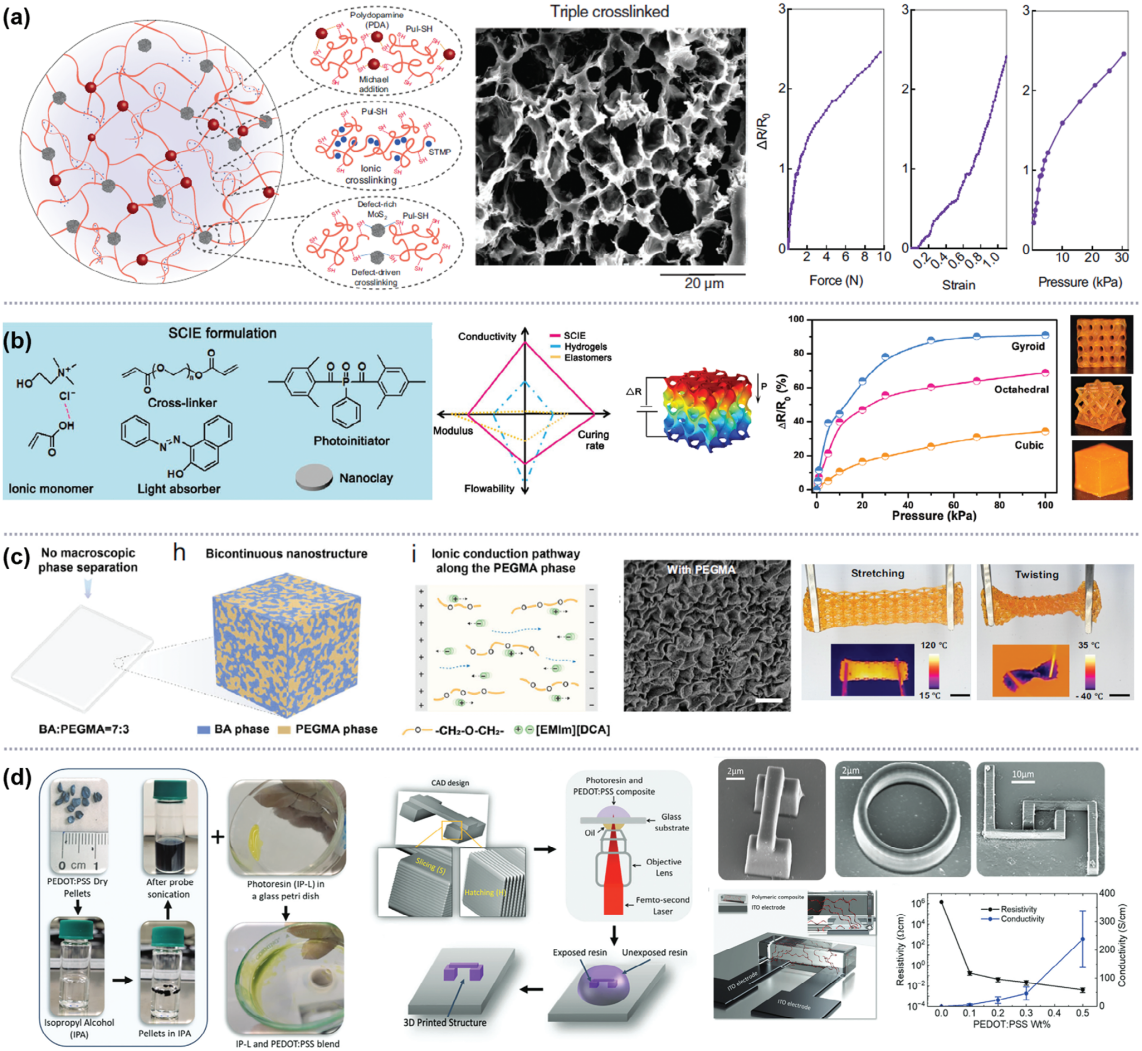
AM processable functional materials for 3D structured sensing electrodes. a) Nanoengineered hydrogel for interconnected porous network formed by triple‐crosslinking. Reproduced with permission.^[^ ^112^ ^]^ Copyright 2024, Wiley. b) Photocurable, solid‐state conductive ionoelastomer for tactile applications. Reproduced with permission.^[^ ^113^ ^]^ Copyright 2022, Wiley. c) Highly conductive and stretchable ionogels with bicontinuous nanostructures. Reproduced with permission.^[^ ^114^ ^]^ Copyright 2024, Springer Nature. d) PEDOT:PSS‐based conductive polymer composite resin for 2PP. Reproduced with permission.^[^ ^115^ ^]^ Copyright 2024, Wiley.

New functional materials for AM techniques have been developed to enhance the sensing performance since not all materials are compatible with AM techniques. Roy, et al. developed nano‐engineered hydrogels, featuring adjustable electronic and thermal biosensing capabilities, that can be processable by DIW to fabricate an electronic skin (**Figure** **7**a).^[^ ^112^ ^]^ The hydrogel ink they developed consisted of thiolated pullulan (Pul‐SH), 2D molybdenum disulphide (MoS_2_), as a crosslinking agent, with enhanced hydrophilicity and crosslinking ability by incorporating sulfur vacancies. To provide wet‐tissue adhesive properties, polydopamine (PDA) nanoparticles were incorporated into the hydrogel, which leaded to the formation of a secondary crosslinking network through a Michael addition reaction between thiols of Pul‐SH and the catechol groups of PDA. A third crosslinking network was introduced by using sodium trimetaphosphate to crosslink the pullulan chains. The interconnected porous network formed by triple‐crosslinking demonstrated in this work offers a highly elastomeric and mechanically stable hydrogel sensing platform for the detection of strain, pressure, and temperature changes in a single sensing platform.

The 3D printed hydrogels/ionogels have great potential for applications such as wearable tactile sensors and bioelectronics, but conventional liquid‐phase hydrogels/ionogels exhibit limited stability in their electrical‐mechanical properties due to the evaporation and leakage of the liquid within hydrogels/ionogels.^[^ ^113^ ^]^ Zhang, et al. developed a fast photocurable, solid‐state conductive ionoelastomer (SCIE) for DLP technique that enables the fabrication of high‐resolution 3D architectures (even ≈50 µm overhanging lattice).^[^ ^113^ ^]^ The SCIE photocurable resin consists of ionic monomers made of acrylic acid and choline chloride complex as a solid‐state ionic skeleton, phenylbis(2,4,6‐trimethylbenzoyl)phosphine oxide as a photoinitiator, poly(ethylene glycol) diacrylate as a cross‐linker, nanoclay as non‐covalent cross‐linker to further improve the mechanical properties of SCIE, and Sudan I as a light absorber (Figure 7b). The printed SCIE‐based building blocks exhibited good Young's modulus (up to 6.2 MPa), stretchability (fracture strain of ≈292%), excellent electrical conductivity tolerance in a wide range of temperatures (from −30 to 80 °C) and fine elasticity and antifatigue ability even after 10 000 cycles at 80% compression. They demonstrated the gyroid lattice‐structured piezoresistive sensor and gap‐based tactile sensor exhibiting 3.7‐ and 44‐fold higher sensitivities than their bulky counterparts, respectively.

He, et al. also developed a UV‐curable, highly conductive and stretchable nanostructured (CSN) ionogel for 3D printed ionotronic sensors.^[^ ^114^ ^]^ They developed a photopolymerization‐induced microphase separation strategy to prepare CSN ionogels with bicontinuous nanostructures (ranging from 5 to 153 nm) comprising interpenetrating benzyl acrylate (BA) and poly(ethylene glycol) methyl ether methacrylate (PEGMA) domains (Figure 7c). When the amount of PEGMA is sufficient, the PEG side chains from PEGMA form continuous microchannels percolating in the polymer network which highly facilitate ion transport of ionic liquid, 1‐ethyl‐3‐methylimidazoium dicyanamide ([EMlm][DCA]). The resultant CSN ionogels achieve high ionic conductivity over 3 S m^−1^, high stretchability (over 1500%), low hysteresis (0.4% at 50% strain), and thermostability from −72 to 250 °C. They demonstrated a capacitive sensor with 3D microarchitectures with high resolution up to 5 µm using DLP.

Amruth, et al. developed a conductive polymeric composite resin for 2PP technique to print miniaturized conductive 3D microstructures that can be integrated into circuits, sensors and biomedical devices.^[^ ^115^ ^]^ PEDOT:PSS is a promising sensing material since it is conductive and responsive to external stimuli such as temperature, humidity, gas, pressure, and strain because of its ionic conductivity and hygroscopic nature. The challenge comes from incompatibility between acrylate‐based photoreactive resin, mostly used for 2PP, and PEDOT:PSS, which hinders the ink stability and printing accuracy. To improve the miscibility between hydrophobic acrylate resin and PEDOT:PSS, they employed a two‐step approach: dissolving dry PEDOT:PSS pellets in a compatible solvent (i.e., isopropyl alcohol) and then mixing with acrylate resin (Figure 7d). This ensures homogenous mixing of acrylate resin and PEDOT:PSS after solvent evaporation. An electrical conductivity of 3.5 × 10^2^ S cm^−1^ was achieved from the 3D printed microstructure (80 µm × 10 µm). They demonstrated an ultra‐fast micro‐3D printed humidity sensor with the highest sensitivity of 0.05%RH^−1^ in the humidity range of 0–80%RH and a response and recovery time of 0.15s and 0.3s, respectively, at 0.2 wt.% of PEDOT:PSS concentration in the polymeric composite.

#### 2.4.2 3D Printed Electrocatalysts

**Figure 8:**
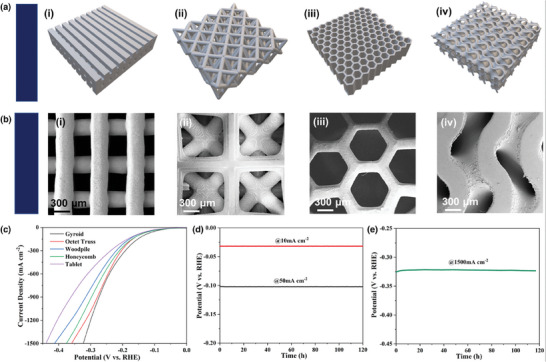
a) 3D models of woodpile (i), octet (ii), honeycomb (iii), and gyroid (iv) structures. b) SEM images of Ni‐Mo electrodes of woodpile (i), octet (ii), honeycomb (iii), and gyroid (iv) lattices. c) HER polarization curve of different temperatures sintered 53 at. % Mo plate electrodes in 1 M KOH solution at 2 mV s^−1^ with 85% iR compensations. d) Anodic current measured at 0 V (vs Hg/HgO) as a function of the scanning rate for 900 °C‐sintered, 1100 °C‐sintered, 1300 °C‐sintered, and 1500 °C‐sintered 53 at. % Mo electrodes. Reproduced with permission from.^[^ ^117^ ^]^ Copyright 2024, Wiley.

3D printed electrodes have been shown to provide opportunities to engineer the mass transport for electrocatalysis. Rocha, et al. demonstrated that TPMS 3D lattices such as gyroid, Fischer–Koch, and Schwarz reduce the limitations on electrocatalyst performance from bubble entrapment in the case of Inconel electrodes (Ni ≈ 60%, Cr ≈ 22%, Mo ≈ 9%, Fe ≈ 4%, Nb ≈ 3%).^[^ ^116^ ^]^ Their experimental electrolysis studies show more than a 20% reduction in overpotential under forced electrolyte flow. Their study also shows that varying the porosity from 50% to 90% engineers the electrochemical surface area (ECSA) but also determines the ability to lower the ohmic resistance of their electrodes via forced electrolyte flow. A suitable test for the claim that 3D electrodes can support efficient mass transport is the operation at high current densities. Xun, et al. have recently reported high current density (1500 mA cm^−2^) catalysts based on monolithic structures of 3D stereolithography printed Ni‐Mo^[^ ^117^ ^]^ (**Figure** **8**). Their structures have hierarchical combinations of macro and nanoscale porosity in their 3D gyroid lattice structures. They also show that the bimetallic NiMo composition yields the lowest overpotential at very high current densities. In their comparison against multiple lattice types such as woodpiles, octet, and honeycomb structures, Xun, et al. find optimal performance from the TPMS gyroid lattices, resulting in an overpotential of just 458 mV to achieve 500 mA cm^−2^ (Figure 8c). The advantages of 3D printed electrocatalysts can be seen in comparison with metal foam electrodes. Metal foams can exhibit challenges regarding bubble accumulation at high current densities. For example, Bleeker, et al. demonstrated that at high current densities metal foam electrodes encountered additional ohmic resistance specifically from bubbles trapped in the foam structure.^[^ ^118^ ^]^ This additional resistance created a direct tradeoff between electrochemical surface area and the ability to evolve bubbles in dense pore structures.

**Figure 9:**
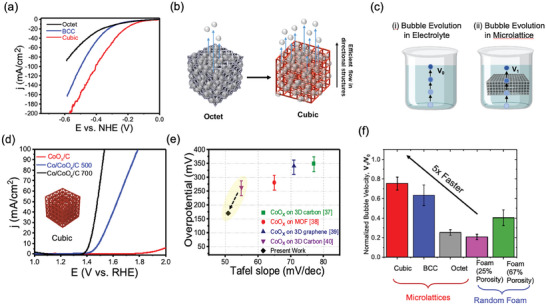
a) Comparison of linear scan voltammetry curves for the HER using cubic, octet, and BCC microlattices coated with Cu/CuO_x_. b) Proposed mechanism of accelerated bubble evolution for microlattices with aligned pores (cubic lattices). c) Bubble evolution velocity measurements normalized by velocity in electrolyte. d) OER performed with cubic microlattices of Co/CoO_x_ fabricated by PIAM. e) Comparison of overpotential and Tafel slope of 3D‐printed Co/CoO_x_ microlattice electrodes for OER. f) Bubble evolution velocity for cubic, BCC, and octet lattices versus random foam structures as measured with high‐speed video microscopy. Reproduced with permission.[^71^] Copyright 2024, Wiley).

Utilizing our method of polymer infusion additive manufacturing of core‐shell microlattices, we have recently demonstrated high performance earth‐abundant catalysts for both hydrogen evolution reaction (HER) and oxygen evolution reaction (OER) based on metal / metal oxide heterostructures of Cu/CuO_x_ and Co/CoO_x_.^[^ ^71^ ^]^ These porous microlattices (**Figure** **9**) exhibit significant enhancements in activity compared with powders of the identical material, which is shown to be due to the mass transport advantages of bubble evolution in ordered 3D structures. Moreover, this work quantitatively compares bubble evolution velocities in 3D microlattices (cubic, octet, and BCC) against random foams, showing a 5× enhancement in bubble speed through cubic lattices. The enhanced mass transport of these 3D microlattice electrodes leads to state‐of‐the‐art performance as well as electrochemical stability, with the optimal Co/CoO_x_ electrodes for OER showing just 1.40 V to achieve 10 mA cm^−2^ and optimal Cu/CuO_x_ microlattice exhibiting 145 mV for HER. The ideal bubble evolving properties of the cubic lattice structure have also been demonstrated in electrodes for the OER using selective laser sintering (SLS) 3D printed nylon scaffolds electroplated with Ni/Cu electrodes by Marquez, et al.^[^ ^119^ ^]^ This study also showed the versatility of 3D printing technologies for investigating various electrode topologies in 2D (cones, pillars, etc.) for promoting bubble desorption. This study by Marquez, in a similar claim to those posed by Tiwari,^[^ ^71^ ^]^ shows through significant (20000×) cyclic voltammetry (CV) cycling that the advantage of 3D lattice structures can be observed in both activity *and* stability.

An alternative to post‐processed polymer 3D scaffolds is to use 3D printed metal electrodes as mechanically and electrochemically stable platforms for designing catalysts. However, studies of these systems have shown that they require surface modification for achieving high activity. For example, Ambrosi and Pumera in 2017 demonstrated 3D printed electrodes consisting of selective laser melting (SLM) fabricated steel electrodes with electrochemically deposited Pt and iridium oxide (IrO_2_)^[^ ^120^ ^]^ coatings. This study performed both HER and OER, showing that large cm‐scale “gauze” structures could provide efficient bubble evolution and that 3D printed polymers could also be utilized for the electrochemical cell structure. While this study did not demonstrate smaller scale high surface area structures, it did reveal the potential for 3D printed metals to be utilized as mechanically and electrochemically robust platforms for electrolysis. In a similar vein, Ambrosi and Pumera have demonstrated that these 3D printed steel electrodes can be coated in earth abundant transition metal compounds such as Ni‐MoS_2_ and NiFe double hydroxides,^[^ ^121^ ^]^ achieving overpotentials of ≈300 mV for HER. Hofer, et al. have also shown that 3D printed titanium scaffolds can be engineered for electrocatalysis by coating of Ir and anodization to increase the electrochemical surface area.^[^ ^122^ ^]^ These 3D printed metal electrodes are capable of stable operation at the 300 mV overpotential level in acidic media, though 100‐h stability studies showed the limitations from dissolution of the noble metal catalyst coatings.

Beyond stainless steel, 3D printed Ni electrodes such as those demonstrated by Xu, et al. in 2023 combine the material advantages of traditional Ni foams with the ability to design 3D periodic structures via stereolithography methods.^[^ ^123^ ^]^ Xu, et al. show detailed video analysis of efficient bubble detachment from their gyroid structures with 100–200 µm pores and performed simulations highlighting how the evolution of smaller bubbles modified electrochemical potential in the electrolyte solution. The superaerophobicity of their structures allowed the demonstration of high current density HER (500 mA cm^−2^) at just 104 mV overpotential. 3D printed aluminum‐based structures fabricated via SLM can also provide a platform for the HER, as shown by Dong, et al.^[^ ^124^ ^]^ “Batwing” shaped TPMS‐type Al lattices were utilized in this work and were coated with NiCl_2_ to enhance activity via formation of Ni nanoparticles. This study also involved a comparison of a wide variety (≈8) of TPMS structures, revealing optimal performance by diamond and gyroid lattices, which were attributed to the ability to achieve turbulent conditions during flow‐through operation.

## 3 Challenges and Opportunities for 3D Electrodes

### 3.1 Accelerating Multiphysics Design

As discussed in Section 2, one of the great promises of AM is its potential for producing customized device geometries for any given application. However, designing custom 3D electrodes involves more than structural optimization for mechanical properties. Instead, the tailoring of electrochemical, electrical, and thermal properties requires a multiphysics consideration of the geometry and the scaling of various material properties with size. A central future challenge for applying AM to complex devices will be how to accelerate and automate this design process. For example, 3D design methods implemented with deep learning and artificial intelligence could be applied toward new objectives that capture physical properties (conductivity, electrochemically active surface area, etc.) beyond mechanical behavior. This challenge has been addressed recently in adjacent research fields regarding the use of artificial intelligence to control the expensive experimental budgets required for optimization of complex devices such as proton exchange membrane fuel cells^[^ ^125^ ^]^ and perovskite solar cells.^[^ ^126^ ^]^ These cases illustrate the need to make tradeoffs between models that simulate the detailed physical properties or alternately treat the devices as a black box described with a set of features of ranked importance. The integration of 3D printed electrodes downstream into more complex devices with many design requirements (porosity, mass, surface area, mechanical stiffness, etc.) may necessitate this black box treatment to efficiently guide and optimize their design. Interestingly, the need for this multiphysics design also extends to the 3D printing process itself. Future opportunities to overcome limits to resolution and printing speed could require integration of components into the 3D part design to allow resin transport, for example, viaducts incorporated to allow infusion for accelerating continuous liquid interface 3D printing.^[^ ^127^ ^]^

The ability to rapidly deploy new electronic devices such as 3D printed batteries or sensors into a 3D printed structure would be a significant step in the move toward *on‐demand* manufacturing with AM. There are various technological opportunities that could arise from having such an agile manufacturing capability for complex systems, for example, in the field of robotics. Soft robotics, as one example, will require integration of conductive electrodes into larger actuator and sensor assemblies that have carefully designed conductive and mechanical properties.^[^ ^128^ ^]^ Additionally, a trend toward smaller dimension robots at the mm‐scale will require that 3D printed material design allow for simultaneous optimization of both mechanical and electrical properties toward cases such as dielectric elastomers.^[^ ^129^ ^]^

### 3.2 Multimaterial Printing

Multimaterial printing remains a grand challenge for AM, and one that is particularly relevant for 3D electrodes given the common demand for both electrically conductive structures and electrochemically functional surfaces in both energy and sensing applications. Multimaterial systems will become essential for leveraging the full capabilities of 3D AM, since 3D porous electrodes also must be integrated into more complex systems. For example, sensors require housings designed to control the introduction of the analyte. As highlighted in recent reviews of this topic, 3D printed microfluidics are a clever extension of this principle to allow “modular” sensors and integration of many devices in parallel.^[^ ^130^ ^]^ Similarly, catalysts require the ability to deliver electrolyte and extract reaction products such as gases. The integration capability has been explored by authors in the field, for example, work by Ambrosi, et al. has shown 3D printed water splitting flow cells that are self‐contained using 3D printed parts.^[^ ^121^ ^]^ In their recent work, Marquez, et al. have highlighted the opportunity for 3D printed flow cells to provide critical information regarding the stability and scalability of new electrocatalyst materials.^[^ ^131^ ^]^ This work shows that 3D printed flow cells can emulate the mass transport that would be present in industrial scale electrolyzer stacks. Additionally, opportunities that could be accessed for 3D printed electrodes in catalysis and synthesis may depend on being able to make the reactor components *themselves* catalytically active. Embedding catalyst materials in the reactor components has been demonstrated by Wei, et al. via metal 3D printing and tuning of the metal feedstock powders.^[^ ^132^ ^]^

### 3.3 Enabling Multifunctional 3D Device Integration

Given the recent advances in 3D printed electrodes, there are opportunities for leveraging the on‐demand nature and the ability to fabricate complex structures beyond the limitations of traditional machining. For electrical and electrochemical devices, this will require rethinking what the fundamental units are for design and assembly. For example, microelectronics adopts a modular paradigm of component integration on circuit boards. This paradigm is fundamentally “2D” and is ill suited to some system‐level applications requiring additional capabilities such as mechanical flexibility or the ability to fill non‐rectangular 3D volumes. Freeform 3D design possible with AM directly addresses these challenges. For example, electrochemical devices need not look identical to those fabricated by traditional manufacturing methods in rectangular pouches or cylindrical rolled geometries. Additionally, electrocatalysts and fuel cells need not necessarily be formed via complex stacks of electrodes and membranes separating machined flow channels.

Multifunction 3D printed devices in the future may also integrate optoelectronic functionality, for example, the ability to emit and detect light distributed across structural, 3D components.^[^ ^133^ ^]^ A slightly more exotic functionality enabled by 3D printed electroluminescent materials could even be imitation of the color matching abilities of a chameleon, as demonstrated by Zhang, et al. in 2022.^[^ ^134^ ^]^ This color matching ability required integration of a closed loop of sensors and light emitting devices of multiple colors in a 3D printed structure (a four legged “ELBot”). This integration of closed loop sensing and control represents an important frontier for the future of 3D printed multifunctional devices.

Toward this goal of sensing in 3D printed devices, there is the possibility for fabrication of complex device arrays in parallel, providing the capacity for multifunctional devices. With the capability to easily fabricate many smaller parallel structures, 3D printed sensors could truly approach some of the promises of the sensing literature, offering the ability to pair various sensing materials and to leverage more powerful statistical methods for the elimination of otherwise confounding cross‐sensitivities. These goals will also require integration with electronics, a challenge that calls for further development of the conductive materials that can interconnect electrochemical devices to microelectronics. Sensors and energy devices require complementary abilities to deliver and extract electrical power. In the case of sensors, instrumentation circuits are also necessary to operate the sensors, read the data, and eventually store and transmit the signals. In the case of energy devices, the 3D electrode is the component that eventually must be paired with electronics for delivering or extracting power. The best opportunities for 3D printed systems to excel relative to incumbent technologies will be in their capacity to simplify design and enhance performance by overturning existing paradigms of integration (particularly those relying on manual assembly).

## 4 Conclusion

This review highlights the transformative potential of 3D printed conductive electrodes at the mesoscale (10 µm–1 mm) using additive manufacturing technologies for sensing and electrochemical applications. We have explored the unique electrical and electrochemical properties of these structures and considered how these characteristics can be optimized through both their microscale 3D geometries and the constituent materials. We specifically consider how post‐processing can transform 3D printed nonconductive lattice structures into conductive electrodes with tunable surface morphologies bridging the nanoscale properties and the mesoscale functionality. However, realizing its full potential requires significant advancements in AM processable functional materials, scalable multimaterial printing techniques, computational design tools, and the integration of 3D printed electrodes into functional devices, as discussed in Section 3.

Creating hierarchical structured electrodes through multimaterial 3D printing processes requires careful consideration of material compatibility, resolution, dimensional accuracy, and integrity of properties across entire structure. Beyond the resolution limit of AM techniques, designing electrodes that bridge the nanoscale to mesoscale using hierarchical materials, such as by incorporating nanomaterials, could lead to further improvements, particularly in applications requiring high surface area and enhanced electrochemical reactivity. Furthermore, the selection of methodologies, sequencing and timing of post‐processing steps in multimaterial printing is crucial for achieving both electrical conductivity and mechanical properties.

The challenges of achieving reproducibility, reliability, and long‐term stability of 3D printed electrodes in dynamic environments remain significant. To address these challenges, further research is required to understand material performance at the interface between 3D printed electrode structures and functional devices. Continuous effort in developing innovative materials, optimizing geometries, and advancing scalable manufacturing techniques will lead to the creation of more efficient, cost‐effective and versatile electrodes, opening up exciting opportunities for next‐generation electronic devices in sensing and electrochemical applications.

## Conflict of Interest

The authors declare no conflict of interest.
